# Cardiogenic Shock Requiring Extracorporeal Membrane Oxygenation Support in a Patient with Panhypopituitarism: A Case Report

**DOI:** 10.7759/cureus.4995

**Published:** 2019-06-25

**Authors:** Denis Huang, Kristin Kreitler, Scott Tilton, Nicholas C Cavarocchi, Hitoshi Hirose

**Affiliations:** 1 Surgery, Thomas Jefferson University, Philadelphia, USA; 2 Emergency Medicine, Thomas Jefferson University Hospital, Philadelphia, USA; 3 Surgery, Critical Care, Penn Presbyterian Medical Center, Philadelphia, USA; 4 Cardiothoracic Surgery, Thomas Jefferson University, Philadelphia, USA

**Keywords:** cardiogenic shock, ecmo, panhypopituitarism

## Abstract

We present a 58-year-old female with a past history of a pituitary adenoma resected two years prior to admission who developed polymorphic ventricular tachycardia and cardiogenic shock requiring veno-arterial extracorporeal membrane oxygenation (VA-ECMO). We noted that the patient had stopped taking all of her medications six months prior to presentation. An extensive workup revealed acute panhypopituitarism with secondary hypothyroidism, secondary adrenal insufficiency, and central diabetes insipidus. She was immediately initiated on thyroid and adrenal hormone replacement therapy as well as fluid replacement. Within five days of her medical treatment, the patient’s cardiac function improved and she was successfully weaned from VA-ECMO and subsequently discharged home with appropriate hormone replacement therapy.

## Introduction

The pituitary gland, consisting of the anterior and posterior lobe, is an important endocrine organ that exerts significant influence over cardiovascular homeostasis. The anterior pituitary gland is responsible for the production of adrenocorticotropic hormone, thyroid-stimulating hormone, growth hormone, follicle-stimulating hormone, luteinizing hormone, and prolactin. These hormones modulate downstream effects through the hypothalamic-pituitary-target organ axis. The posterior lobe produces vasopressin and oxytocin, which are secreted directly from nerve terminals. The most common causes of pituitary insufficiency are a consequence of surgical resection and radiation treatment of pituitary adenomas [1]. Deficiencies of pituitary hormones can clinically manifest as secondary hypothyroidism, adrenal insufficiency, hypogonadism and central diabetes insipidus. These patients often require hormone replacement therapy to maintain adequate thyroid, adrenal, sex hormones levels and to avoid dehydration secondary to diabetes insipidus. Dysregulation of thyroid hormone, growth hormone and adrenal hormones is classically linked with an increased risk and severity of cardiovascular disease [2]. Central diabetes insipidus may add further complications due to intravascular volume loss and stimulation of the sympathetic nervous system.

Generally, it is more common to see presentations of chronic heart failure in the setting of individual hormone insufficiencies compared to more severe hypopituitarism. The progression of heart disease also tends to be chronic, due to pathologic remodeling and compensation. In many patients, cardiac function improves under hormone replacement therapy alone. It is rare to have acute collapse of the cardiovascular system resulting from panhypopituitarism that requires mechanical circulatory support, such as venous-arterial extracorporeal membrane oxygenation (VA-ECMO). Here we present, to the best of our knowledge, the first case report of panhypopituitarism resulting in cardiogenic shock requiring VA-ECMO support.

## Case presentation

A 58-year-old female (height 170 cm, body weight 65 kg, body surface area 1.7 cm^2^) presented to the outside hospital’s emergency department complaining of shortness of breath and “hot flashes”. Her past medical history was significant for a pituitary adenoma, which had been resected two years prior. Electrocardiogram (EKG) on presentation showed sinus bradycardia (55 beats per min) with frequent premature ventricular ectopy and prolonged QT intervals (550 msec). Echocardiogram revealed a dilated left ventricle with an ejection fraction 10% with right ventricular dysfunction. She underwent cardiac catheterization and no significant coronary disease was identified. Cardiac index was measured at 1.2 L/min/kg and, as a result, she was placed on norepinephrine and dobutamine infusions for hemodynamic support. During catheterization, the patient acutely decompensated, developing hypoxia and mental status change. Arterial blood gas drawn at the outside hospital recorded a PaO2 of 37 mmHg with O2 saturation 69% on a non-rebreathing facemask. She required emergent intubation and was admitted to the cardiac care unit (CCU) at the outside institution for further management. While in the CCU, she continued to develop unstable ventricular tachycardia refractory to medical management, including the administration of multiple anti-arrhythmic medications and defibrillation. She was hypotensive with a mean arterial pressure of 55 mmHg, requiring high dose of norepinephrine (0.6 mic/kg/min) and dobutamine (10 mic/kg/min). She was also hypoxic requiring 100% FiO2 to maintain appropriate oxygen saturation. Due to hemodynamic instability, she was placed on VA-ECMO via bilateral groin access and transferred to our institution for further management.

Repeated echo at our institution showed cardiac standstill (Video 1).

**Video 1:** Echocardiography shortly after ECMO before medical treatment showed cardiac standstill. ECMO: Extracorporeal membrane oxygenation

A routine admission laboratory panel revealed hypocalcemia, elevated lactate, hyperglycemia with a normal renal and hepatic functions (Table 1). Shortly after admission, she was noted to be producing large amounts of urine. Her urine output was measured at 8 liters over the first 12 hours at our institution. Initially, VA-ECMO flow was restricted to 2-2.4 L/min, most likely due to hypovolemia. Aggressive fluid replacement therapy was initiated and ECMO flow improved to 4.5 L/min. A subsequent endocrine panel was abnormal (Table 1).

**Table 1:** Admission laboratory panel.

	Patient’s levels	Reference
Hematology data		
White blood cell count	6.5 B/L	4–11 B/L
Platelet	69 B/L	140–400 B/L
Hemoglobin	9.3 g/dL	12.5–15 g/dL
Chemistry data		
Sodium	144 mmol/L	135–146 mmol/L
Potassium	4.6 mmol/L	3.5–5 mmol/L
Bicarbonate	19 mmol/L	21–26 mmol/L
Creatinine	0.9 mg/dL	0.7–1.4 mg/dL
Calcium	6.6 mg/dL	8.5–10.3 mg/dL
Lactate	2.4 mmol/L	0.5–2 mmol/L
Glucose	53 mg/dL	70–100 mg/dL
Endocrine data		
Thyroid stimulating hormone (TSH)	0.34 IU/mL	0.3–0.5 IU/mL
Parathyroid hormone (PTH)	347 pg/mL	11–67 pg/mL
Free T3	1.4 pg/mL	2.0–4.4 pg/mL
Free T4	0.3 ng/dL	0.7–1.7 ng/dL
Random cortisol	2.7 mg/dL	16–20 mg/dL
Morning cortisol	4.6 mg/dL	16–20 mg/dL

Endocrine and nephrology were consulted to assist with the diagnosis and treatment plan. Based on the history of pituitary resection, as well as the discovery that the patient was noncompliant with her medications for approximately six months prior to presentation, it was believed her numerous endocrine abnormalities and cardiogenic shock were secondary to acute panhypopituitarism. Initial treatment included intravenous fluid replacement for central diabetes insipidus, intravenous levothyroxine for hypothyroidism (50 mcg daily), corticosteroids for adrenal insufficiency (100 mg every eight hours), and calcium replacement (1000 mg three times a day). As her fluid balance normalized, her lactic acidosis eventually resolved.

After three days of ECMO support and medical treatment, a repeat transthoracic echocardiogram (TTE) confirmed improving left and right ventricular function. An ECMO weaning trial with decreased ECMO flow was conducted on ECMO day 4 and she was decannulated from ECMO support on ECMO day 5. Inotropic support was not required after post-decannulation. Echocardiography after ECMO removal showed a left ventricular ejection fraction of 45% and normal right ventricular function (Video 2).

**Video 2:** Echocardiography after ECMO removal showed improved biventricular function. ECMO: Extracorporeal membrane oxygenation

After being successfully weaned from ECMO therapy, the patient was transitioned to oral prednisone (5 mg daily) for adrenal insufficiency, oral levothyroxine (100 mcg daily) for hypothyroidism, and intravenous calcitriol (1 mcg daily) and oral calcium carbonate (1,000 mg three times daily) for hypocalcemia. She was extubated and liberated from mechanical ventilation on post-decannulation day 3. She was then transferred back to the referral facility on post-decannulation day 7 and eventually discharged to a rehabilitation facility and home without recurrence of heart failure symptoms.

## Discussion

Our patient, with a history of pituitary adenoma status post pituitary resection, presented with endocrine abnormalities suggestive of panhypopituitarism. She also exhibited signs of acute collapse of cardiac function in the setting of severe hypotension refractory to fluid resuscitation and vasopressor support. Her diagnosis was supported by her noncompliance with taking her medications and constellation of cardiovascular compromise. She initially required VA-ECMO support due to her hemodynamic instability and initiated on hormone replacement therapy. We believe this is the first report of cardiogenic shock requiring ECMO therapy secondary to acute panhypopituitarism. Furthermore, our observations of her clinical course provide evidence of the important role that the pituitary gland plays in maintaining proper cardiovascular status.

The role of thyroid hormone in cardiovascular function is well documented and studied. Any level of abnormal thyroid function is classically linked to chronic heart failure and poor outcomes following myocardial infarction by affecting myocardial remodeling and causing continued deterioration of organ function [3,4]. Hypothyroidism often leads to the disruption of diastolic left ventricular function and, in time, is associated with decreased chronotropy, decreased cardiac contractility and cardiac output and increased peripheral vascular resistance [5]. The constellation of symptoms also includes QT-prolongation and ventricular arrhythmias [6]. Ultimately, chronic hypothyroid status can result dilated cardiomyopathy [7,8].

While there is considerable evidence supporting the pathophysiological role of thyroid hormone in chronic heart failure, there are comparatively few cases of thyroid deficiency contributing to acute heart failure or cardiogenic shock. Ashwath et al. describe one patient with history of flutter who presented with cardiogenic shock that was precipitated by AV nodal re-entrant tachycardia secondary to previously undiagnosed primary hypothyroidism [9]. Dharmasena et al. describe another patient, with no history of heart disease, who presented with global hypokinesia due to uncontrolled known primary hypothyroidism [10]. Both cases present with hypotension refractory to fluid resuscitation and vasopressor support, and then have rapid stabilization of blood pressure after administration of thyroxin. Similarly, both patients experience full recovery to their baseline cardiac function once placed on proper hormone replacement.

Adrenal insufficiency is another endocrine-related cause of cardiac disease. Dysfunction can occur at any level of the hypothalamic-pituitary-adrenal gland axis and can cause both acute and chronic heart failure. Shimizu et al. describe a 54-year-old female who developed cardiogenic shock due to acute central adrenal insufficiency [11]. Koga et al. describe a 62-year-old male who presented with congestive heart failure due to adrenal insufficiency and was diagnosed with empty sella syndrome [12]. Acute reversible cardiomyopathy is also associated with primary adrenal insufficiency, or Addison’s disease [13,14]. Similar to the effects of prolonged hypothyroidism, hypotension is a common manifestation of adrenal insufficiency that can precede further cardiovascular compromise.

In addition to its effects on fluid homeostasis, posterior lobe hormone such as vasopressin also modulates peripheral vascular resistance and cardiac output. Ohara et al. describe a 63-year-old male with left ventricular dysfunction with undiagnosed central diabetes insipidus [15]. The case report notes that central diabetes insipidus may predispose patients to heart failure, particularly those who already have a history of heart disease. For these patients, tight control of water and sodium intake is recommended in addition to desmopressin therapy to prevent progression of heart failure.

Acute heart failure secondary to hypopituitarism, though less prevalent than those with individual hormone etiology, has also been reported. The exact mechanisms of hypopituitarism-related cardiogenic shock are not clear but likely related to the combined effects of multiple endocrine insufficiencies. In literature, the most common presentations are postpartum cardiomyopathy induced by anterior pituitary failure, where heart failure has developed insidiously underneath undiagnosed Sheehan’s syndrome [16,17]. While initial presentations are impressive, each respective patient saw complete if not considerable improvement of cardiac function following proper hormone replacement therapy using a combination of corticosteroid and levothyroxine. The extent of recovery is associated with the severity of hypopituitarism and length of time prior to the start of proper treatment.

Cardiogenic shock due to endocrine organ hypofunction requires significant initial medical intervention but improves with proper hormone replacement therapy. Rarely, does the severity of cardiac compromise require more invasive procedures to be performed. Lohiya et al. report a 19-year-old patient with autoimmune adrenal insufficiency who was empirically treated with a wide range of inotropes and interventions including intra-aortic balloon pump for cardiogenic shock [18]. Krishnamoorthy et al. report a 21-year-old male who required ECMO for acute cardiomyopathy due to fulminant Addison’s disease [19]. While ECMO is traditionally used to support complications of cardiopulmonary arrest, it has also been used during endocrine emergencies. However, indications have been restricted to pheochromocytoma crisis and thyroid storm [20].

To the best of our knowledge, no other case with cardiogenic shock requiring ECMO support has been described in the setting of sustained panhypopituitarism. Our patient’s cardiomyopathy and left ventricle ejection fraction (LVEF) of 10% likely developed during the six months of uncontrolled panhypopituitarism. During her medication noncompliance, she developed a perfect storm of endocrinological derangements that led to her sudden hemodynamic collapse and cardiac arrhythmias. Prompt initiation of ECMO therapy, prior to diagnosis, likely saved her life and prevented permanent damage to her cardiopulmonary system.

## Conclusions

This case emphasizes the use of VA-ECMO therapy as a feasible option for hemodynamic support in cases of cardiogenic shock of unknown etiology. ECMO maintains hemodynamic stability and end organ perfusion, providing invaluable time for further workup to be performed. Although clinically rare, panhypopituitarism and other forms of endocrine dysfunction should be considered on the differential during initial history-taking in a patient with cardiogenic shock where the exact cause is not clear. The workup involves simple laboratory panels which allow providers to avoid more invasive testing, such as myocardial biopsy. Furthermore, proper treatment can be quickly initiated to ensure preservation and recovery of cardiac function.
